# Comparison of body composition between fashion models and women in general

**DOI:** 10.20463/jenb.2017.0032

**Published:** 2017-12-31

**Authors:** Sunhee Park

**Affiliations:** 1. Department of Modeling, Dongduk Women´s University, Seoul Republic of Korea

**Keywords:** fashion model, anthropometry, body mass index, waist-to-hip ratio, skinfold thickness

## Abstract

**[Purpose]:**

The present study compared the physical characteristics and body composition of professional fashion models and women in general, utilizing the skinfold test.

**[Methods]:**

The research sample consisted of 90 professional fashion models presently active in Korea and 100 females in the general population, all selected through convenience sampling. Measurement was done following standardized methods and procedures set by the International Society for the Advancement of Kinanthropometry. Body density (mg/ mm) and body fat (%) were measured at the biceps, triceps, subscapular, and suprailiac areas.

**[Results]:**

The results showed that the biceps, triceps, subscapular, and suprailiac areas of professional fashion models were significantly thinner than those of women in general (p<.001), and that their waist size was also significantly smaller (p<.001). However, hip circumference showed no significant difference. Body mass index, waist-to-hip ratio, and body fat (%) in professional fashion models were significantly lower than those in women in general (p<.001), while the body density in professional fashion models was significantly greater (p<.001).

**[Conclusion]:**

Body density in professional fashion models is higher, due to taller stature, than in women in general. Moreover, there is an effort on the part of fashion models to lose weight in order to maintain a thin body and a low weight for occupational reasons.

## INTRODUCTION

Professional fashion models are thought to exemplify bodily perfection and become symbols of ideal beauty for the general public.^1^^-^^2^ By taking a leading part in popular culture, professional fashion models are mediators between fashion and the public; as such they influence the public and become objects of envy.^3^^-^^4^ Moreover, the fact that a tall stature and thin body are necessary conditions for modeling, requiring continual attention by designers and individual models, cannot be overlooked.^5^^-^^6^

Research performed outside of Korea and reported by the World Health Organization in 1998 showed that 66% of professional fashion models were underweight and that around 1/4 of American fashion models had a body mass index (BMI) below 17.5, the cutoff for anorexia defined by the American Psychiatric Association. ^7^^-^^9^ Review of international research reveals a diversity of positions. Some state that the body type of the fashion model is ideal and beautiful,^10^^-^^11^ while others claim that female models with a waist-to-hip ratio (WHR) of 0.7 are the most attractive and that they set the standard for appeal; still others list professional fashion models among the groups affected by eating disorders.^12^^-^^15^ This diversity shows that professional fashion models are subject to social pressure to maintain a thin or ideal body type. 

Research in Korea focused mainly on the following: the choice of specialization by professional fashion models, the level of satisfaction about their choice, their career awareness, their awareness about specialization and their choice of a professional path through national modeling agencies, the state of the management industry, the diversification of the profession, and historical changes and trends affecting male and female fashion models.^16^^-^^23^

Yoo reported that professional fashion models have low muscle mass, longer limbs, and low weight, but high body fat (%). However, this research was done conducted not on real models but on aspiring models such as students in modeling schools. Therefore, the conclusions cannot be directly applied to presently active fashion models.^24^ A later study applied vague criteria to differentiate between highly successful professional fashion models and general fashion models, thus making the results unreliable.^25^

Thus, as concern for their health is increasing and since they serve as standards of physical beauty for the general public, there is a need to broaden the research on body composition of Korean professional fashion models. The present study aimed to quantify the body types of professional fashion models, and thus to foster a healthy body image and help correct a dysfunctional occupational attitude. 

## METHODS

### Research subjects

The research sample was obtained by employing convenience sampling to select 90 professional fashion models presently active in Korea and 100 women in the general population. Fashion models were selected from among those with more than one year of professional experience and participating in an average of 10 or more yearly regular fashion shows. The average professional experience of the sample was 5 years and 7 months (±3 years and 2 months). Women in general were selected among Seoul residents aged 20 to 30 years old. The physical characteristics of the sample are presented in Table 1. 

**Table 1:** Physical characteristics of professional fashion models and general females

	FM (n=90)		Con (n=100)		p-value
	M ± SD	Range	M ± SD	Range	
Age (year)	24.4 ± 3.2	18 - 33	23.0 ± 2.4	20 - 28	0.001
Weight (kg)	54.3 ± 3.1	46 - 63.4	53.5 ± 6.2	41.1 - 73.5	0.236
Height (cm)	177.9 ± 2.0	174.4 - 182.4	161.2 ± 5.4	148.2 - 174.7	0.001

FM: Professional fashion model, Con: female control

### Research procedures and methods

Prior to any measurement, all participants were advised of the purpose of the research and consent was obtained and confirmed through a written form. Adhering to the standards set by the International Society for the Advancement of Kinanthropometry (ISAK), body measurements were taken twice. An average was obtained based on measurements on the right side of the body. Whenever an error higher than ±2% was registered, a third measurement was taken. In these cases, the median value was taken as a representative value. 

For the measurement method, we followed the guidelines of the ISAK.^26^ Sites chosen for subcutaneous body fat measurement included biceps, triceps, subscapular, and suprailiac regions, using conversion formulas ^27^ as follows:

1. BMI = weight (kg)/height (m)²
2. WHR = waist (cm)/hip (cm)
3. Body density (20-29 years old) = 1.1599-(0.0171 × L)
*L is the log of the sum of four skinfold measurements.
*The DW equation is age dependent.

Means and standard deviations were obtained by processing the data collected throughout the research using Microsoft Excel, while the comparison between professional fashion models and women in general was performed using SPSS 20.0 for Mac. An independent t-test was used to compare the two groups and the significance level (α) was set at 0.5. 

## RESULTS

### Comparison between body measurements in professional fashion models and women in general

Table 2 shows the comparison between body measurements in professional fashion models and women in general. Professional fashion models had significantly thinner biceps, triceps, subscapular, and suprailiac areas than women in general (p<.001). However, there was no significant difference in hip circumference (p>0.05). 

**Table 2:** Professional fashion models and general females body measures comparison

Skinfold site (mm)		FM (n=90)		Con (n=100)		p-value
		M ± SD	Range	M ± SD	Range	
Thickness (mm)	Biceps	6.8 ± 2.4	0.3 – 1.5	8.7 ± 3.3	0.3 – 2.0	0.001
	Triceps	12.6 ± 2.7	0.5 – 1.8	16.2 ± 3.9	0.7 – 2.6	0.001
	Subscapular	11.2 ± 2.6	0.6 – 2.0	16.7 ± 5.0	0.7 – 3.0	0.001
	Suprailliac	11.8 ± 3.6	0.5 – 2.5	15.9 ± 5.0	0.7 – 2.9	0.001
Circumference (cm)	Waist	62.2 ± 2.6	5.7 – 7.1	66.3 ± 6.2	5.0 – 9.6	0.001
	Hip	89.7 ± 2.4	8.3 – 9.7	90.5 ± 4.5	8.2 – 10.1	0.157

FM: professional fashion model, Con: female control

### Comparison between body composition in professional fashion models and a control group

Table 3 compares the body composition in professional fashion models with that of a control group of women in general. Professional fashion models had significantly lower BMI, WHR, and body fat (%) than women in general (p<.001), whereas body density was significantly higher (p<.001). 

**Table 3:** Professional fashion models' and general females' body composition compared

	FM (n=90)		Con (n=100)		p-value
	M ± SD	Range	M ± SD	Range	
BMI	17.1 ± 0.9	14.5 - 19.6	20.6±2.1	15.9 - 26.6	0.001
WHR	0.69 ± 0.03	0.63 - 0.78	0.73±0.06	0.59 - 1.06	0.001
Body density (mg/mm)	1.04381± 0.00617	1.03040 - 1.06365	1.03463 ± 0.00788	1.01810 - 1.05845	0.001
Body fat (%)	24.2±2.8	15.4 - 30.4	28.5±3.6	17.7 - 36.2	0.001

FM: Professional fashion model, Con: female control

As professional fashion models gain importance in the fashion industry, the need for an appropriate approach to their health becomes more significant. The following conclusions were drawn. 

First, the comparison between professional fashion models and women in general indicated that, with the exception of hip circumference, all other areas were significantly thinner in fashion models (p<.001). 

Second, the BMI, WHR, and body fat (%) in professional fashion models were significantly lower (p<.001), while body density was significantly higher (p<.001). 

Thus, we verified that the body composition of professional fashion models was lower in subcutaneous fat than that of women in general and that their BMI, WHR, and body fat (%) was lower than average. A close association was observed in fashion models between dieting for weight loss to meet occupational requirements and low weight and subcutaneous fat levels. 

## DISCUSSION

The present research is the first comparative study on body composition in professional fashion models and Korean women in general. The purpose was to provide information on body measurements and composition as baseline data for use in selection of healthier models as well as for education. 

There have been a limited number of studies on body type in fashion models and most have used photographic material or self-reporting rather than data based on actual measurements.[25, 28] BMI can be calculated without actual measurement, but the values may not be accurate. Similarly, WHR values determined from photographic material can be imprecise. 

The skinfold test employed in this research uses calipers to measure subcutaneous fat. This test was reported to be inefficient due to its dependence on the skills of the measurer and other technical issues.^30^ However, it is widely used among researchers because it can be used in any setting and is easy to perform.[30, 31]

In addition, even though several methods can be used to determine body fat (%) from measured values, the method employed in the present study is highly reliable because different formulas are used, depending on the sex and age of the research subjects.[27, 31]

Other than hip circumference, the results showed that there was a significant difference between the two groups in all other areas (p<.001). These results prove that professional fashion models have very low levels of subcutaneous fat in relation to the general female population. We conclude that fashion models are obliged by their profession to look thin before the public and that their thin body type is a result of excessive dieting. 

On the one hand, the BMI of professional fashion models appeared significantly lower than that of women in general (p<.001), whereas their body density appeared to be significantly higher (p<.001). This may reflect the fact that model heights are considerably greater and that BMI is lower in models than in women in general. We found that the BMI in models of 17.1±0.9 was lower than the BMI of 17.5 set by the American Psychiatric Association as a cut-off for anorexia. Korean national standards classify a BMI lower than 18.5 kg/ ㎡ as underweight, 18.5-22.9 kg/㎡ as normal, and 23- 24.9 kg/㎡ as overweight or risk weight.[8, 32] The fact that the BMI of professional fashion models is below underweight seems to indicate that, responding to occupational demands, fashion models follow severe dieting regimes in order to maintain a thin body type. However, some have stated that professional fashion models are not at risk of anorexia because they adjust to the thin body and low weight that is a necessary occupational condition.^15^ Moreover, the American Council on Exercise has stated that differences in body composition can reflect differences in race, culture, age and sex, leading to the conclusion that fashion models cannot be considered a risk group simply because of their thin body type.^33^

The WHR of 0.69 (±0.03) in models in our study is significantly lower than that of women in general (p<.001). Tovee et al. compared the BMI and WHR in 300 British fashion models with those in the general population and their finding of a WHR of 0.71 (±0.02) in fashion models is similar to that in our study.^28^ Singh indicated that men feel more attraction when a woman's WHR is 0.7 or lower, and our study leads us to conclude that the WHR of 0.69 of Korean models makes their body type attractive by international standards.[ 13] Professional fashion models train themselves to continuously center the balance of their bodies using abdominal strength and repeatedly practice walking on high heels. Moreover, due to the characteristics of their profession, they follow continuous diets and in general maintain strict regimens, so that the above measurements may reflect these efforts. 

Body fat (%) in fashion models was 24.2±2.8, significantly lower than that in women in general (p<.001). This result is also low with respect to the value of 25-32% that the American Council on Exercise has indicated as appropriate for women in general. Jeong analyzed the body types of Korean fashion models and hinted at the possibility of professional fashion models being skinny-fat, which is verified when the muscle volume is low with respect to the amount of body fat.^25^ However, since the body fat calculation method based on the skinfold test can only predict body density and body fat percentage, the results are insufficient to determine the possibility of skinny-obesity.[25, 33] Therefore, there is still a need to verify the body fat(%) using either bioimpedance analysis or dual energy X-ray absorptiometry. 

Similar to dancers, fashion models stand on the stage and communicate feelings not through words but through the movement of their bodies.^34^ However, in order to highlight their clothing, they are required to work hard on maintaining a thin body type. Dancers are asked to keep restrictive diets to maintain their body types because they stand on the stage and entertain the public by emphasizing their physical appearance.^35^^-^^36^ However, dancers need to exert their muscles in order to stage an impressive performance, and usually accompany their dieting regimen with necessary training. By contrast, the reality of professional fashion models is that they focus exclusively on food restriction rather than accompanying it with physical exercise. Therefore, fashion models should combine dieting with physical exercise. Professional fashion models receive the attention of the public and are an object of envy for women. Instead of simply employing starvation diets, correct maintenance of body type requires a diet based on adequate nutritional education and the development and application of a training program that responds to the professional needs of fashion models. 

Professional fashion models not only raise the purchasing desires of the public but also create new fashion and thus can influence trends. For this reason, a study on the physical and biological characteristics of professional fashion models seems to be of value to both the models and the general public. 

As a suggestion for future research, studies should focus on professional fashion models as a special occupational cluster and should use a variety of methods for the development of body fat (%) equations, including bioimpedance analysis and dual energy X-ray absorptiometry. The results may show differences in BMI and body composition between normal women and fashion models. Future research will also need to consider the importance of a healthy body type and objective and correct awareness about body types. 
